# Intraflagellar transport complex structure and cargo interactions

**DOI:** 10.1186/2046-2530-2-10

**Published:** 2013-08-14

**Authors:** Sagar Bhogaraju, Benjamin D Engel, Esben Lorentzen

**Affiliations:** 1 Department of Structural Cell Biology, Max-Planck-Institute of Biochemistry, Am Klopferspitz 18, Martinsried, D-82152, Germany; 2 Department of Molecular Structural Biology, Max-Planck-Institute of Biochemistry, Am Klopferspitz 18, Martinsried, D-82152, Germany

**Keywords:** Intraflagellar transport, Cilium, IFT, IFT complex, IFT cargo

## Abstract

Intraflagellar transport (IFT) is required for the assembly and maintenance of cilia, as well as the proper function of ciliary motility and signaling. IFT is powered by molecular motors that move along the axonemal microtubules, carrying large complexes of IFT proteins that travel together as so-called trains. IFT complexes likely function as adaptors that mediate interactions between anterograde/retrograde motors and ciliary cargoes, facilitating cargo transport between the base and tip of the cilium. Here, we provide an up-to-date review of IFT complex structure and architecture, and discuss how interactions with cargoes and motors may be achieved.

## Review

Twenty years ago, Kozminsky and colleagues first described intraflagellar transport (IFT) as a motility in the *Chlamydomonas* flagellum that is distinct from flagellar beating [1]. IFT trains were observed by electron microscopy to be linear arrays of electron-dense particles spanning the distance between the outer doublet microtubules and the flagellar membrane. Following the discovery of IFT, biochemical purification of native IFT complexes from *Chlamydomonas* revealed 15 polypeptides that organize into two IFT sub-complexes, known as IFT-A and IFT-B [2,3]. IFT polypeptide orthologues were also found in mice [4,5], suggesting that IFT proteins are largely conserved. Subsequent studies identified additional IFT proteins, bringing the current IFT protein count up to 20 [5-11]. Mutations in IFT proteins have been shown to cause several ciliopathies [12-22]. The genetic deletion of an entire IFT protein often leads to a general defect in cilia assembly (presumably due to IFT complex disruption), making it difficult to assess the specific functions of individual IFT proteins from mutant phenotypes alone [8,23-31]. Thus, a more complete understanding of IFT protein function in ciliogenesis, including cargo and motor interactions, will require detailed molecular and structural studies of IFT complexes. Structural investigations of IFT complexes have been limited so far to electron tomographic reconstructions of IFT particles *in situ*[32] and the high-resolution crystal structure of the IFT25/27 sub-complex [33]. However, the overall architecture of the IFT complex is starting to take shape, largely as a result of biochemical studies [25,26,34,35]. In this review we attempt to partition IFT proteins into principal domains (PD) and auxiliary domains (AD) based on the current literature. Whereas PD mutations lead to IFT complex destabilization with general ciliogenesis phenotypes, AD mutations may facilitate the study of specific IFT protein functionality. Such a division may assist in designing experiments to probe the roles of individual IFT proteins in cilium formation and function.

### The intraflagellar transport complex: a protein-protein interaction platform?

Bioinformatic analysis of IFT proteins predicts a large number of potential protein-protein interaction domains such as tetratrico peptide repeats (TPRs), WD40 β-propellers and coiled-coils [36-39]. Strikingly, with the exception of the two small GTPases IFT22 and IFT27, none of the other IFT proteins are predicted to have enzymatic activity. The prediction is thus that the IFT complex forms a large platform with multiple protein interaction sites that allows binding to molecular motors as well as ciliary cargoes.

**Figure 1:**
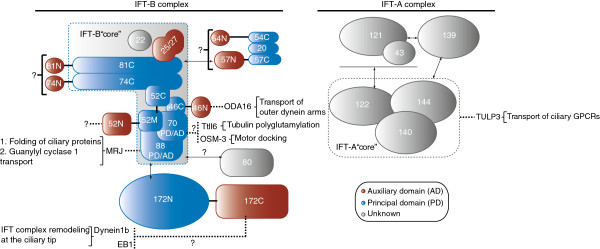
**Domain organization and known cargo interactions of intraflagellar transport complex proteins.** Intraflagellar transport (IFT) proteins are divided into distinct modules, referred to in this review as principal domains (PDs) and auxiliary domains (ADs), serving principal structural (blue) and auxiliary interaction (red) roles, respectively. Proteins for which there may not be a clear boundary between PD and AD are labeled as “PD/AD”. The probable interacting cargoes of various IFT ADs are indicated with a dashed line. The ADs of IFT81, IFT74, IFT52, IFT54 and IFT57 still remain to be characterized. All of the IFT proteins are abbreviated as the numerical part of their names. The letters N, M and C next to the numbers refer to the N-terminal, middle and C-terminal domains of the corresponding protein. IFT-A proteins, IFT80 and IFT22 are colored grey because their associations with other IFT proteins and ciliary cargoes are poorly characterized. EB1, End binding protein 1; GPCR, G-protein coupled receptor; MRJ, Mammalian relative of DNAJ; ODA, outer dynein arms; OSM, Osmotic avoidance abnormal protein; Ttll6, Tubulin tyrosine ligase-like 6; TULP3, tubby like protein 3.

Structure prediction using the HHpred server [40] revealed that most IFT proteins likely contain multiple domains [39]. Limited proteolysis on *in vitro* reconstituted IFT complexes demonstrated that only a subset of these domains are required for IFT complex formation, indicating that numerous domains are available to interact with other binding partners such as ciliary cargoes or motors [35]. Most IFT proteins can therefore be divided into PDs and ADs as described above (Figure 1). The main function of PDs is to provide structural stability, and thus they are well conserved in protein sequence to ensure the integrity of IFT complex formation. However, most IFT protein domains not required for IFT complex stability (the ADs) are also highly conserved in sequence, likely reflecting important functions such as ciliary cargo interactions. A good example of the PD/AD division is IFT46, a core component of IFT-B, where only the IFT46 C-terminal domain is required for the stability of the IFT complex via interaction with the C-terminal domain of IFT52 [25,35], while the N-terminal domain is involved in the ciliary transport of outer dynein arms (ODAs) [24,41,42]. Similarly, IFT52 interacts directly with at least four different IFT proteins (IFT74/81, IFT46, IFT70 and IFT88) via its middle and C-terminal domains, while the conserved N-terminal domain is not required for IFT-B complex formation and thus likely constitutes an AD [25,35]. The N-terminal domain of IFT74 is also not required for IFT-B core complex formation and may constitute an AD [35]. The peripheral IFT proteins IFT54 and IFT57 both have predicted coiled-coil domains at the C-termini that interact with IFT20 [43-45]. However, the N-terminal regions of both IFT57 and IFT54 are predicted to be alpha helical domains that could constitute ADs [39] (Figure 1).

It is important to note that while the PD/AD boundary of some IFT proteins is well defined, this is not the case for all IFT proteins. In particular, TPR domain-containing proteins such as IFT70 and IFT88 may possess a single structural module that functions as both a PD and an AD (Figure 1). Another example is IFT25 and the small GTPase IFT27, which form a stable heterodimer that can be considered as a single structural module [33]. While the IFT25/27 heterodimer directly binds the “core” IFT74/81 complex [35], it also contains a conserved surface patch in close proximity to the GTPase active site of IFT27 that may interact with a yet unidentified binding partner in a nucleotide-state-dependent manner [33]. Interestingly, IFT25 knockout mice show no ciliogenesis defects but die at birth due to sonic hedgehog (Shh) signaling dysfunction [46]. This indicates that the IFT25/27 sub-complex is not needed for the stability of the IFT complex and may function in the IFT of Shh signaling components. Additionally, IFT25 and IFT27 are not present in *Caenorhabditis elegans* and *Drosophila melanogaster*[10,38]. Thus, IFT25/27 may be defined as an AD module (Figure 1).

### Ciliary targeting sequences

Proteins that localize to subcellular compartments such as mitochondria or the nucleus have distinct sequence motifs (known as cellular ZIP codes) that specifically target them to their respective organelles [47]. Although the cilium is topologically equivalent to the cytoplasm, there are transition zone structures at the ciliary base that prevent random diffusion of both soluble and membrane-bound macromolecules into the cilium [48-56]. As approximately 600 different proteins reside within the cilium [57], it seems likely that one or more ZIP codes also exist for ciliary targeting [58].

#### The (F/Y/W)R motif

One of the earliest reports of a ciliary targeting sequence (CTS) was the identification of a phenylalanine-arginine (FR) motif in the C-terminal cytoplasmic regions of *C. elegans* olfactory receptor proteins ODR-10 and STR-1 [59]. Deletion of this FR motif from ODR-10 and STR-1 resulted in dispersed localization of the receptors in the cell body, indicating that the FR motif is required for ciliary localization. This (F/Y/W)R motif is conserved in several ciliary G-protein coupled receptors (GPCRs) including somatostatin receptor 3 (SSTR3), serotonin receptor 6 (5-HTR6) and rhodopsin, suggesting a widely prevalent and conserved mechanism of targeting GPCRs to the cilium [59]. A similar motif in mammalian Smoothened (smo) was also shown to be required for localization to the cilium [60]. However, several GPCRs that contain (F/Y/W)R motifs do not localize to cilia, indicating that the ciliary targeting of GPCR proteins is more complex. Inspection of the rhodopsin crystal structure reveals that an equivalent residue (F313 of alpha-helix VIII) [61], identified to be a part of the (F/Y/W)R motif in the other GPCRs, is buried within the hydrophobic core of the protein and hence may be necessary for proper protein folding. This suggests that mislocalization of ciliary GPCRs upon mutation of the (F/Y/W)R motif may be an effect of compromised structural integrity of the GPCR fold rather than a primary defect in ciliary targeting. It is thus not surprising that different CTSs have been identified in several ciliary GPCRs including SSTR3, 5-HTR6 and rhodopsin, as described in the following sections.

#### The Ax(S/A)xQ motif

Comparative sequence conservation analysis of ciliary and non-ciliary GPCRs revealed a different consensus amino acid sequence, Ax(S/A)xQ (where x denotes any amino acid), in the third intracellular loop of ciliary GPCRs [62]. Mutating the conserved A or Q in this motif resulted in the mislocalization of SSTR3 and 5-HTR6 [62]. Conversely, the chimeric non-ciliary GPCR Htr7 with this motif inserted into its third intracellular loop showed markedly increased ciliary localization [62]. Recently, a similar signal sequence was found in the third intracellular loop of another ciliary GPCR, melanin-concentrating hormone receptor 1 [63]. These results indicate that the Ax(S/A)xQ motif is both necessary and sufficient for the localization of these GPCRs. The mouse GPCR Gpr161 was also shown to contain a CTS ((I/V)KARK) in its third intracellular loop that is both necessary and sufficient for localization to cilia [64]. Interestingly, this CTS is different from the Ax(S/A)xQ motif described above, suggesting that the third intracellular loops of different GPCRs may contain distinct sequence motifs that confer ciliary localization.

#### The VxPx motif

In addition to the (F/Y/W/)R motif described above, rhodopsin was shown to contain a VxPx motif at its cytoplasmic C-terminus that serves as a CTS [65,66]. The Ca^2+^ ion channel polycystin-2 (PC2) also has an N-terminal RVxP motif that is required for its ciliary localization [67], and polycystin-1 (PC1), a direct interacting partner of PC2, contains a similar CTS (KVHPSST) at its cytoplasmic C-terminus [68]. Thus, PC1, PC2 and rhodopsin share a common (K/R/Q)VxPx motif required for ciliary localization.

#### The KRKK NLS-like motif

Compared to the CTSs of membrane proteins, very little is known about the ciliary targeting of soluble proteins. Although there is increasing evidence that tubulin, ODAs and retrograde dynein motors are IFT cargoes [24,69,70], it is unknown how these soluble proteins are recognized by the IFT machinery. Recently, however, the KRKK motif was identified as a CTS in the C-terminal tail of the homodimeric anterograde IFT motor KIF-17 [71]. Remarkably, this CTS is very similar to the nuclear localization signal (NLS) recognized by importin-β2. Both importin-β2 and a Ran-GTP gradient, which are key to nucleo-cytoplasmic transport, also appear to be required for ciliary entry of KIF-17 [71,72]. Furthermore, retinitis pigmentosa 2 was also shown to depend on interaction with importin-β2 for ciliary entry [73]. Another study identified certain nucleoporins at the base of the cilium by immunofluorescence and immunogold electron microscopy [51], although this result remains to be verified. It is unclear at this point whether NLS-mediated ciliary entry is applicable to a broad range of other ciliary proteins.

#### How are ciliary targeting sequences recognized by the intraflagellar transport machinery?

Several lines of evidence suggest that many of the above mentioned membrane proteins are transported into the cilium as IFT cargoes. Rhodopsin requires transport into the outer segment (OS) of photoreceptor cells via the connecting cilium [74], and mutations in IFT proteins or motors have been shown to affect the transport of rhodopsin, indicating a critical role for IFT in this process [4,43,75,76]. The ciliary membrane Transient Receptor Potential Vanilloid (TRPV) channels OSM-9 and OCR-2 undergo IFT-like movements within the cilia of *C. elegans* sensory neurons [77], and a fraction of *Chlamydomonas* PC2 also undergoes directed movement that is likely driven by IFT [78]. Furthermore, in *Chlamydomonas* IFT has been shown to be physically coupled to the movement of flagellar membrane glycoproteins in a Ca^2+^-dependent manner [79]. However, a direct link between the CTSs of membrane proteins and their association with the IFT complex has not yet been demonstrated.

Mutations in IFT-A proteins are known to affect the transport of several membrane proteins including certain ciliary GPCRs [64,80,81]. The BBSome is a multi-protein complex associated with IFT that is also required for the traffic of several membrane proteins into and out of the cilium [82-87]. Interestingly, the domain organizations of the BBS proteins and the IFT-A proteins closely resemble those of the canonical membrane coating complexes (COPI, COPII and Clathrin) [37,38]. Despite being involved in different intracellular trafficking pathways, all of these complexes contain numerous predicted WD-40 β-propeller and TPR/α-solenoid-like domains, suggesting that these systems evolved from a common ancestral trafficking machinery and may utilize similar transport mechanisms [37,38,83]. Intriguingly, in the case of clathrin-mediated vesicular transport, WD40 β-propeller domains are known to selectively bind unique cargo peptides [88]. It is possible that the WD40 β-propeller domains in the IFT-A complex and BBSome selectively interact with the CTSs of ciliary membrane proteins to facilitate their transport into the cilium. Further studies characterizing the IFT-A and BBSome WD40 β-propeller domains may yield insights into ciliary membrane protein targeting and traffic.

### Intraflagellar transport complex-cargo interactions

Although several studies have provided indirect evidence for the association of the IFT complex with ciliary cargoes, proof of direct interactions between IFT proteins and cargoes remains scarce. One of the earliest pieces of evidence for an association between the IFT complex and ciliary cargo comes from the co-immunoprecipitation of IFT74 and IFT139 performed on the soluble fraction of *Chlamydomonas* flagella, which revealed that the IFT complex interacts with ciliary precursors such as dynein light chains, radial spokes, motors and tubulin [89]. Tubulin, a basic structural component of the axoneme, was also shown to undergo IFT-like movement in *C. elegans* sensory neurons [69]. In the following sections we discuss the various reports describing direct and indirect associations between IFT proteins and ciliary cargo.

#### IFT88

Several studies suggest interactions between the TPR-protein IFT88 and ciliary cargo. Co-immunoprecipitation of IFT88 from retinal extracts revealed an association with rhodopsin [90]. Furthermore, IFT88 and rhodopsin were shown to undergo similar movement within the cilia of hTERT-RPE1 cells, indicating that IFT likely plays a direct role in the transport of rhodopsin into the OS of photoreceptor cells [75]. Yeast two-hybrid studies and *in vitro* pulldown assays identified a Dnaj member co-chaperone, MRJ, as a direct interacting partner of IFT88 [90]. GST-tagged MRJ was also shown to associate with the photoreceptor-specific membrane protein guanylyl cyclase 1 (GC1) in a co-immunoprecipitation from bovine retinal extracts. This interaction was further confirmed by *in vitro* pulldown experiments using GST-MRJ and a HIS-tagged cytosolic fragment of GC1. It is possible that MRJ aids in the transport of ciliary GC1 by serving as an adaptor between GC1 and IFT88. As an IFT cargo, MRJ may also cooperate with HSP70 in the folding of ciliary proteins. The mode of interaction between IFT88 and these potential ciliary cargoes is currently unknown.

#### IFT70

IFT70 is another protein in the IFT complex that is predicted to contain TPR structure. Available evidence suggests that DYF-1, the *C. elegans* orthologue of IFT70, is required for the association of IFT particles with the IFT motor OSM-3. Two motors in *C. elegans*, heterotrimeric kinesin-2 (also called kinesin-II) and homodimeric OSM-3, coordinate to drive anterograde transport [91,92]. While both kinesin-2 and OSM-3 propel IFT in the middle segment of the cilium at a speed of 0.7 μm/s, OSM-3 alone drives IFT in the distal segment of the cilium at an increased speed of 1.2 μm/s [91,93]. OSM-3 mutants (*osm-3*) were defective in distal segment formation, while the speed of anterograde IFT in the middle segment decreased from 0.7 μm/s to 0.5 μm/s [91,93]. Interestingly, *dyf-1* mutants exhibited a similar phenotype to *osm-3* mutants and lacked OSM-3 movement, indicating that IFT70/DYF-1 is involved in docking IFT particles onto OSM-3 motors [93]. An additional study revealed that OSM-3 is in an auto-inhibitory state *in vitro* and hypothesized that interaction with IFT proteins is required for activation [94]. Surprisingly, however, purified DYF-1 did not activate OSM-3 *in vitro*[94], and it has been suggested that additional factors may be required [23].

The depletion of the zebrafish IFT70 orthologue, fleer, resulted in the loss of axonemal tubulin polyglutamylation and ultrastructural defects of the outer doublet microtubules (MTs) [95]. Expression of only the N-terminal catalytic domain (residues 1 to 505) of the TTLL6 tubulin polyglutamylase enzyme also resulted in the loss of axonemal polyglutamylation but, intriguingly, basal body tubulin in these cells remained polyglutamylated [95]. Thus, it is possible that the C-terminus of TTLL6 directs ciliary localization through an interaction with IFT70. As polyglutamylation is known to affect the function of motors *in vivo*[96-98], it is possible that the OSM-3 motor is sensitive to the loss of tubulin polyglutamylation, and hence the effect of IFT70 on OSM-3 transport activity could be an indirect one [95]. Direct interaction studies between IFT70, OSM-3 type motors and the TTLL6 enzyme will likely shed light on this relationship.

#### IFT46

IFT46 is a well studied IFT-B core protein with an assigned function in the IFT of ODAs [24,41]. A *Chlamydomonas* insertional null IFT46 mutant showed reduced levels of other IFT complex proteins and flagellar assembly defects, indicating that full length IFT46 is necessary for the stability of the IFT complex [24]. A partial suppressor mutation, presumably expressing a C-terminal fragment of the IFT46 protein, alleviated most of the flagellar assembly phenotypes caused by the full depletion of IFT46, restoring wild-type IFT protein levels and normal flagellar length [24]. However, electron microscopy revealed that the axoneme of this suppressor mutant specifically lacks ODAs. This indicates that the N-terminus of IFT46 is involved in the transport of ODAs, while the C-terminus is required for the stability of the IFT complex [24]. This notion is supported by sequence alignments of IFT46 proteins, which only show high sequence identity for the N-terminal part of IFT46 from organisms with motile cilia, likely a reflection of this domain’s conserved role in ODA transport (data not shown). It was later observed that IFT46 directly binds to ODA16, an adaptor protein that bridges the IFT complex with ODAs [41,42]. Further molecular characterization of the IFT46-ODA16-ODA complex is necessary to understand how IFT46 and ODA16 specifically recognize ODAs as ciliary cargoes.

#### The IFT-A complex

Compared to the IFT-B complex, proteins of the IFT-A complex are not well characterized. Co-immunoprecipitation of LAP-tagged tubby like protein 3 (TULP3) from human RPE1 cell extract revealed that IFT-A proteins interact directly with TULP3 [80]. This interaction was further mapped to the IFT-A “core” complex that contains IFT140, IFT144 and IFT122 [80]. Interestingly, depletion of either IFT-A “core” components or TULP3 caused mislocalization of certain ciliary GPCRs. TULP3 contains a TUBBY domain at its C-terminus that binds phosphoinositides [99]. A TULP3 mutant (TULP3KR) that is defective in phosphoinositide binding affected the localization of ciliary GPCRs but was still able to interact with the IFT-A complex [80]. This indicates that TULP3 bridges the IFT-A complex and ciliary GPCRs, thus aiding in ciliary GPCR transport. Further studies are needed to dissect the TULP3 interaction with IFT-A, as well as the specific role of the TUBBY domain in recognizing ciliary GPCRs [64,100].

### Intraflagellar transport complex-motor interactions

The interactions between IFT motors and IFT complexes are central to understanding how the bidirectional movement of IFT trains is regulated, particularly at the IFT turnaround zones at the ciliary base and tip [101]. As described above, IFT70 (with the help of additional factors) may mediate OSM-3 docking to IFT particles in *C. elegans*. However, the binding interactions between IFT complexes and the most evolutionarily conserved IFT motors, heterotrimeric kinesin-2 and cytoplasmic dynein 2, remain more elusive. Kinesin-2 appears to bind the IFT-A complex in *C. elegans*, and is only physically coupled to IFT-B and OSM-3 via the BBSome [93]. Co-immunoprecipitation experiments in vertebrate cells implicated IFT20 and IFT57 in binding kinesin-2 [45,102], and yeast two-hybrid analysis showed that IFT20 strongly interacts with both IFT57 and the KIF3B motor subunit of kinesin-2 [45]. However, these interactions were not verified in an independent study [5]. Tomographic reconstructions of *in situ* IFT particles revealed densities that are most likely kinesin motors connecting IFT particles to the axoneme, but the study did not identify which IFT proteins bind to the motors [32]. Live-cell fluorescence microscopy of IFT in *Chlamydomonas* has indicated that kinesin-2 (or at least the non-motor KAP subunit) may detach from IFT particles at the ciliary tip [103-105]. In contrast, kinesin-2 was observed to undergo retrograde transport in *C. elegans*[106]. If kinesin-2 does separate from IFT particles at the ciliary tip, this implies that kinesin-2 may ensure that only one type of motor is active at a time by inhibiting dynein 2 function during anterograde transport.

In *Chlamydomonas*, co-immunoprecipitation of IFT172 showed an interaction with cytoplasmic dynein 2 that was independent of IFT-A [107], and studies of temperature-sensitive mutants revealed that IFT172 is required for entry of dynein 2 into the flagellum [107,108]. Rescue of *Tetrahymena* IFT172 knockout cells with C-terminally truncated IFT172 constructs resulted in partial recovery of ciliary assembly and accumulation of IFT proteins at the ciliary tips, reminiscent of a retrograde IFT defect [109]. Thus, IFT172 may be divided into an N-terminal PD that binds the IFT-B “core” and a C-terminal AD that interacts with dynein 2 (Figure 1). Additionally, co-immunoprecipitation of the microtubule plus-end-tracking protein EB1 from *Chlamydomonas* flagellar extract pulled down IFT172 independent of both IFT-A and IFT-B [110,111], although it is unknown which domain of IFT172 mediates this interaction. If binding EB1 modulates the affinity of IFT172 to either dynein 2 or the IFT-B “core”, this could contribute to the regulation of IFT turnaround at the ciliary tip. Interestingly, the partial depletion of dynein 2 from mutant *Chlamydomonas* flagella resulted in a compensatory increase in flagellar EB1 [112], so the two proteins may affect each other’s interaction with IFT172.

### The BBSome: bridging IFT-A and IFT-B?

Purification of native IFT particles from *Chlamydomonas* flagella revealed that IFT-A and IFT-B complexes are loosely associated [2]. Despite their weak association *in vitro*, components of IFT-A and IFT-B move together in *C. elegans* sensory cilia, indicating that additional factors may play a role in the IFT complex stability *in vivo*[93,113]*.* Surprisingly, in *C. elegans bbs-7* and *bbs-8* mutants, the components of IFT-A and IFT-B are carried at different speeds by the heterotrimeric kinesin-2 and homodimeric OSM-3 motors, respectively [93]. This suggests that the BBSome, in addition to its well established function in cycling membrane proteins through cilia [82,84,114-117], may also play a role in holding IFT-A and IFT-B together *in vivo*[93]. This conclusion led to the proposal of the “mechanical competition” model, where the BBSome keeps the IFT-A and IFT-B complexes together resulting in IFT that proceeds at an intermediate speed [92]. However, two observations do not agree with the “mechanical competition” model. First, in *Chlamydomonas* flagella, the levels of BBS proteins are substoichiometric compared to IFT protein levels, and the BBSome component BBS4 undergoes IFT with only a subset of IFT particles [82]. Second, in *C. elegans*, a DYF-2 (IFT144 orthologue) point-mutation resulted in the accumulation of BBSomes at the base of the cilium and the absence of BBSomes inside the cilium, but IFT-A and IFT-B complexes nevertheless moved together at intermediate speeds that were similar to wild-type [113]. Interestingly, in the *dyf-2* mutant, IFT-B components failed to associate with the retrograde IFT machinery and thus accumulated at the ciliary tip. These observations led to the proposal of a model where the BBSome plays a role in the formation of stable IFT complexes at the base and the tip of the cilium but is not necessary for IFT complex stability during anterograde IFT [113]. In any case, both models suggest that the BBSome interacts with components of both the IFT-A and IFT-B complexes. Interestingly, in mice, BBSome component BBS1 was shown to directly interact with the IFT-A component WDR19 (IFT144 orthologue) [113]. As for the IFT-B complex, a large scale yeast two-hybrid study with *C. elegans* proteins revealed an interaction between the IFT-B accessory protein DYF-3 and the BBSome component BBS-7 [118]. Further studies are necessary to understand the regulatory role of the BBSome in IFT.

## Conclusions

Although much is known about the overall architecture of the IFT complex and the role of IFT proteins in ciliary assembly and maintenance, molecular details concerning the distinctive roles of the 20 IFT proteins are still elusive. As pointed out in this review, it is likely that many IFT proteins possess principal domains required for IFT complex formation and auxiliary domains used to interact with ciliary cargo and motors. Functional dissection of these domains will remain the focus of extensive research in the coming years. While it is likely that highly abundant ciliary proteins such as tubulin, dynein arms and radial spokes have unique binding sites on the IFT complex, other ciliary cargo may compete via their CTS for binding to more generic cargo sites. In summary, the IFT complex contains numerous TPR and WD40 repeat domains that are expected to fulfill the task of selectively transporting a large number of ciliary proteins. Obtaining direct evidence for these interactions by means of either structural or functional studies would be a significant leap forward for the IFT field.

## Abbreviations

5-HTR6: serotonin receptor 6; AD: auxiliary domains; CTS: ciliary targeting sequence; GC: guanylyl cyclase; GPCR: G-protein coupled receptors; IFT: intraflagellar transport; NLS: nuclear localization signal; ODA: outer dynein arms; OS: outer segment; PC: polycystin; PD: principal domains; Shh: sonic hedgehog; SSTR3: somatostatin receptor 3; TPR: tetratrico peptide repeat; TRPV: Transient receptor potential vanilloid; TULP3: tubby like protein 3.

## Competing interests

The authors declare that they have no competing interests.

## Authors’ contributions

BDE wrote the “IFT complex-motor interactions” section. SB and EL wrote the rest of the manuscript. All the authors read and approved the final manuscript.
